# Supplementary figures and images for: Community carriage of ESBL-producing Escherichia coli and Klebsiella pneumoniae: a cross-sectional study of risk factors and comparative genomics of carriage and clinical isolates
Source: mSphere. 2023 Jun 12;8(4):e00025-23. doi: 10.1128/msphere.00025-23 (PMC10470604; doi:10.1128/msphere.00025-23)

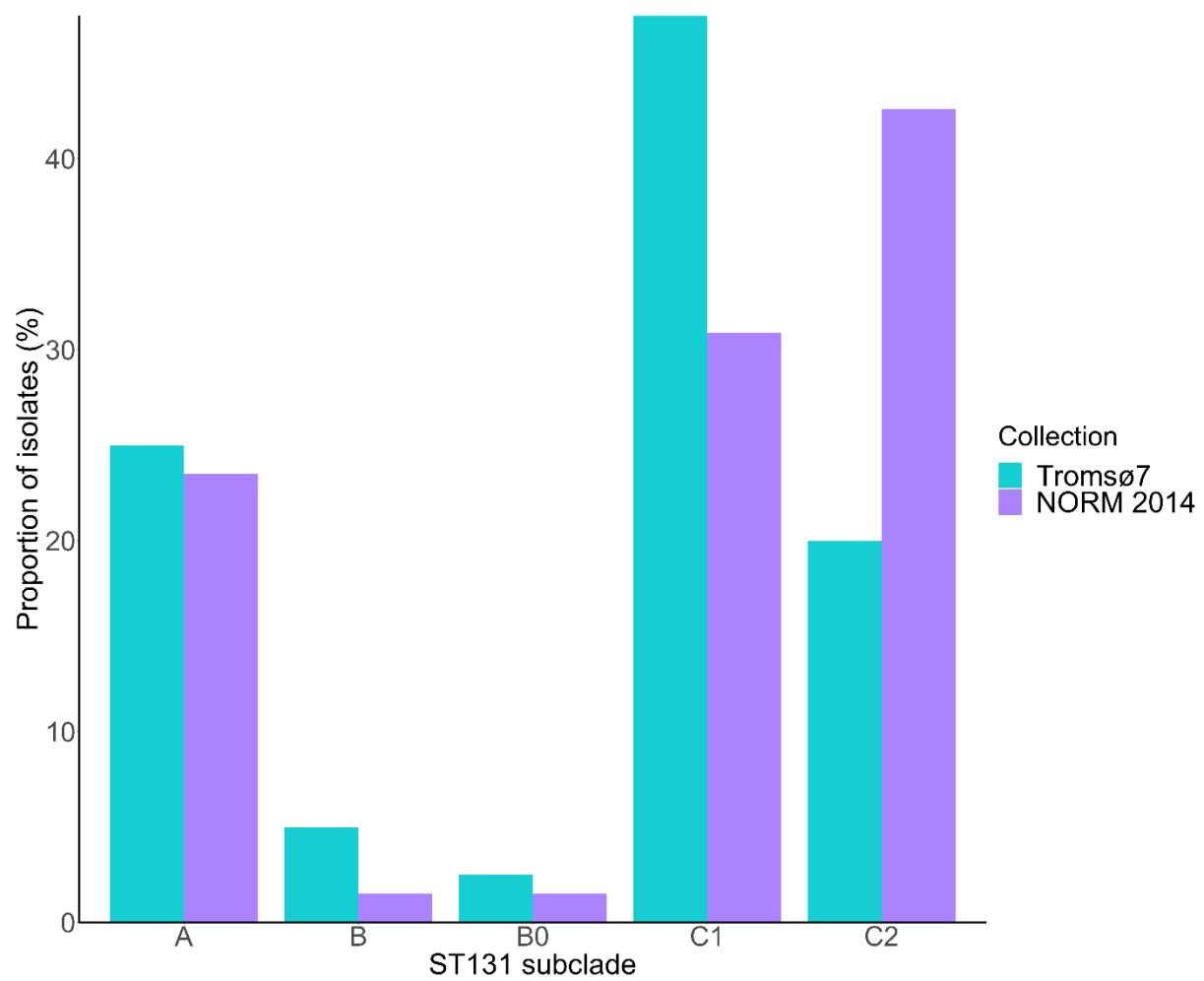

**Supplementary Figure 1**

Supplement: Fig. S1 — ST131 clade distribution among ESBL-E. coli carriage isolates from Tromsø7 (n=40) and clinical isolates from NORM 2014 (n=68). [file msphere.00025-23-s0001.pdf]

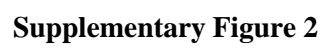

### Supplementary Figure 2

Supplement: Fig. S2 — Replicon type distribution among ESBL-E. coli from carriage isolates from Tromsø7 (n=166) and clinical isolates from NORM 2014 (n=118). [file msphere.00025-23-s0002.pdf]
