# Supplementary material for: Community carriage of ESBL-producing Escherichia coli and Klebsiella pneumoniae: a cross-sectional study of risk factors and comparative genomics of carriage and clinical isolates
Source: mSphere. 2023 Jun 12;8(4):e00025-23. doi: 10.1128/msphere.00025-23 (PMC10470604; doi:10.1128/msphere.00025-23)
Supplement: Fig. S3 — Directed acyclic graph (DAG) illustrating the causal relationship between ESBL-E. coli (ESBL-Ec) gastrointestinal carriage (outcome) and relevant covariates among 4,999 participants in Tromsø7. The variable ‘drug use’ includes antibiotic use past 14 days and acid suppressive medication past four weeks. To set up the most plausible causal relationship between covariates and the outcome, we first searched the literature for relevant factors associated with ESBL-E. coli gastrointestinal carriage. Thereafter, the DAG guided the selection of the multivariable logistic regression model, which was adjusted for the minimal sufficient adjustment set comprising age, drug use, hospitalization, travel abroad, and traveler`s diarrhea (i.e. the variables constituting a confounding pathway). Controlling for the minimal sufficient adjustment set warrants that confounding paths are blocked in order to minimize bias of the causal relationship. The model was not adjusted for sex due to no statistically significant sex difference in prevalence of ESBL-E. coli carriage and because it does not constitute biasing paths after adjustment. For the variables sex and alcohol consumption, a direct effect on ESBL-E. coli carriage is not described or investigated, however they are known to affect the microbiota composition. Hence, intestinal microbiota was included as an unobserved mediator of the causal relationship. [file msphere.00025-23-s0003.pdf]

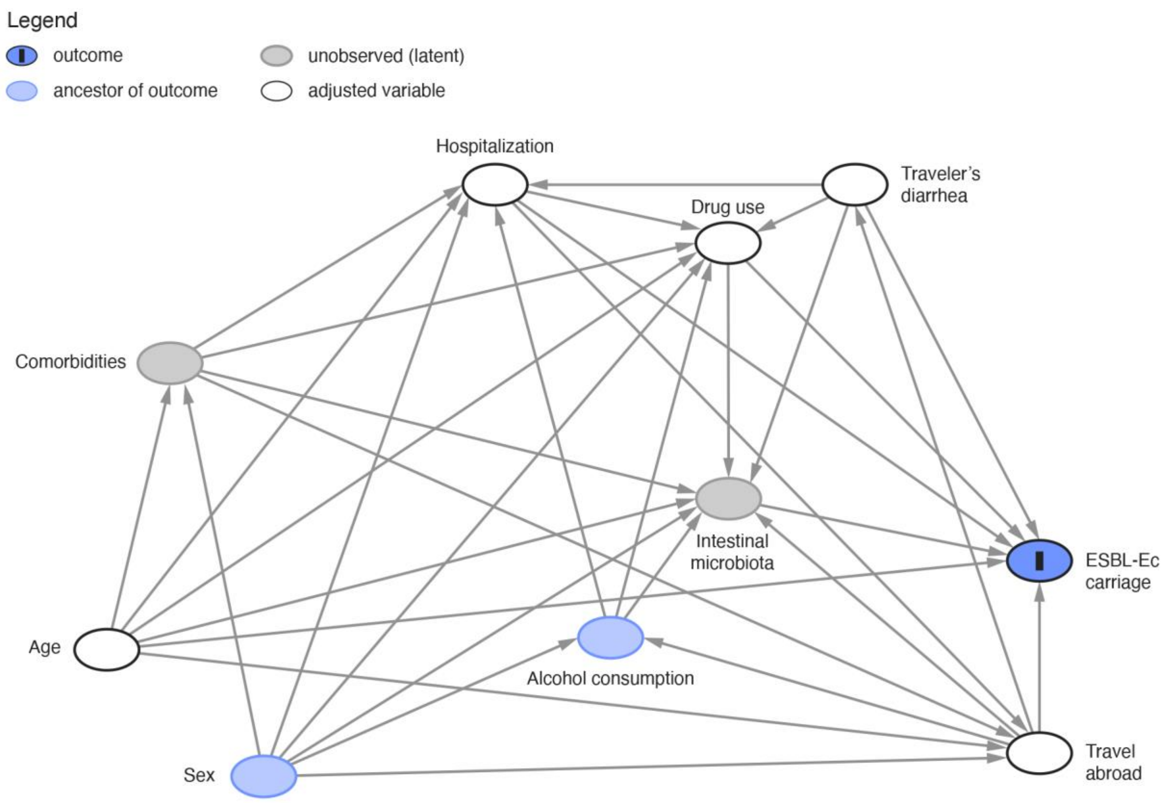

Supplementary Figure 3
