# Supplementary material for: Community carriage of ESBL-producing Escherichia coli and Klebsiella pneumoniae: a cross-sectional study of risk factors and comparative genomics of carriage and clinical isolates
Source: mSphere. 2023 Jun 12;8(4):e00025-23. doi: 10.1128/msphere.00025-23 (PMC10470604; doi:10.1128/msphere.00025-23)
Supplement: Fig. S4 — Directed acyclic graph (DAG) illustrating the causal relationship between K. pneumoniae species complex (Kp) gastrointestinal carriage (exposure), ESBL-E. coli (ESBL-Ec) gastrointestinal carriage (outcome), and relevant covariates among 2,973 participants in Tromsø7. The variable ‘drug use’ includes antibiotic use past 14 days and acid suppressive medication past four weeks. K. pneumoniae is a common cause of healthcare associated infections often combined with antimicrobial resistance. In the literature and our previous study (Raffelsberger N, Hetland MAK, Svendsen K, et al. Gastrointestinal carriage of Klebsiella pneumoniae in a general adult population: a cross-sectional study of risk factors and bacterial genomic diversity. Gut Microbes. 2021 Jan-Dec;13(1):1939599.), we identified several factors associated with K. pneumoniae gastrointestinal carriage, which are overlapping with those associated with ESBL-E. coli carriage. To capture the relevance of K. pneumoniae carriage as an exposure, we included this variable into the causal relationship with ESBL-E. coli carriage. The DAG was used for the selection of variables for the multivariable logistic regression model, to conceptualize confounding and to identify the minimal sufficient adjustment set. Although sex and alcohol consumption represent ancestors of exposure and outcome, the adjustment for age, drug use, hospitalization, travel abroad, and traveler`s diarrhea controls for relevant confounders and blocks biasing paths. The absence of red arrows in the DAG implies that there are no open unadjusted confounding pathways. [file msphere.00025-23-s0004.pdf]

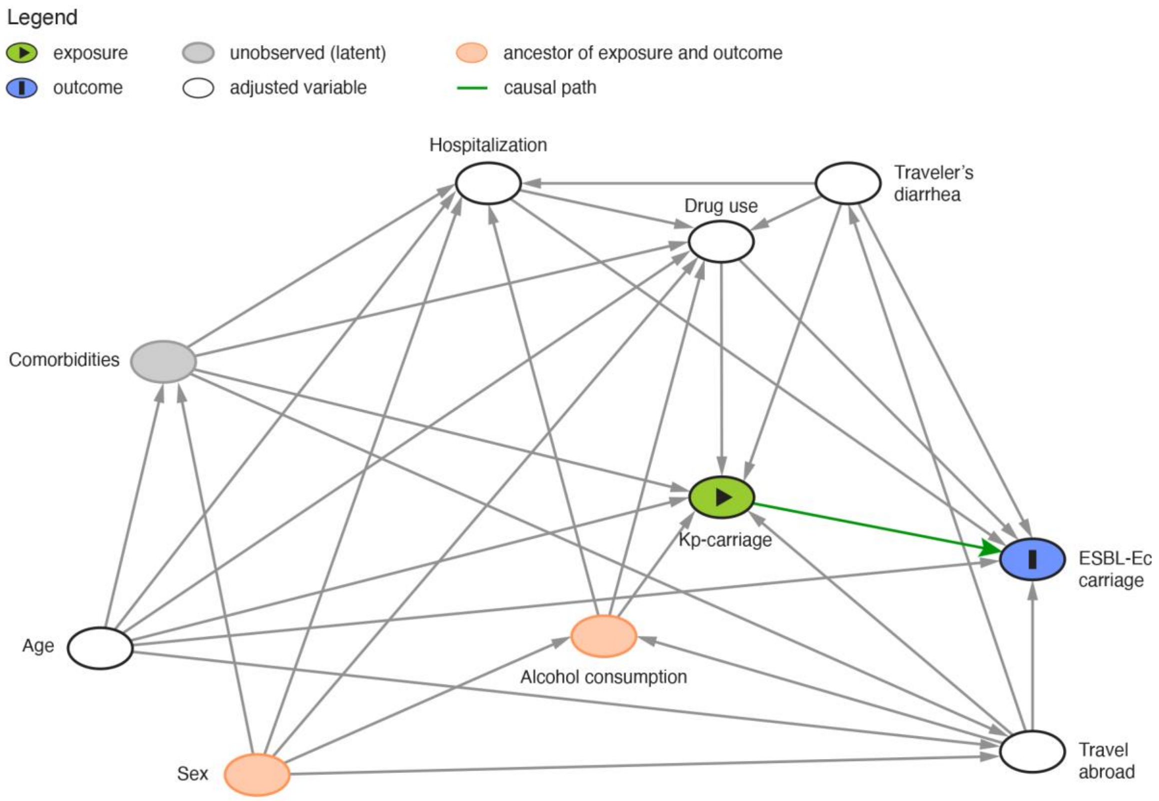

Supplementary Figure 4
