# Supplementary material for: Community carriage of ESBL-producing Escherichia coli and Klebsiella pneumoniae: a cross-sectional study of risk factors and comparative genomics of carriage and clinical isolates
Source: mSphere. 2023 Jun 12;8(4):e00025-23. doi: 10.1128/msphere.00025-23 (PMC10470604; doi:10.1128/msphere.00025-23)
Supplement: Table S1 — Characteristics of the study population. [file msphere.00025-23-s0005.pdf]

Supplementary Table 1. Characteristics of the Study population (n = 4,999) in Tromsø7, 2015-2016.

	Study population	
Characteristics	N	%
Sex		
Men	2,296	45.9
Women	2,703	54.1
Age (years)		
40-49	605	12.1
50-59	814	16.3
60-69	2,128	42.6
70-84	1,452	29.0
Household income		
Low	2,015	42.7
High[a]	2,699	57.3
Current daily smoking		
No	4,360	88.0
Yes	595	12.0
Alcohol consumption frequency		
Never to ≤monthly	1,624	32.7
2-4/month	1,820	36.6
2-3/week or more	1,527	30.7
Hospitalization past 12 months		
No	4,344	88.0
Yes	593	12.0
Antibiotic use past 14 days[b]		
No	4,832	96.9
Yes	155	3.1
Acid suppressive medication last 4 weeks		
No	3,762	79.7
≤weekly	403	8.5
Every week, but not daily	260	5.5
Daily	297	6.3
Travel abroad past 12 months[c]		
No	2,145	44.5
Other regions (excl. Asia)	2,214	45.9
Asia exclusively or Asia + other regions	462	9.6
Traveler`s diarrhea past 12 months[d]		
No	4,724	96.6
Yes	166	3.4

[a] ≥551 000 NOK (€ 53 976/year as per June 2022)
[b] Have you taken any antibiotics (tablets or oral suspensions, nasal ointments, eye drops, or eye ointment) during the past 14 days?
[c] Traveled outside the Nordic countries >1 week duration in the past 12 months.
[d] For each travel abroad past 12 months, the participants were asked if they did experience diarrhea in connection with the travel.
