# Supplementary material for: Community carriage of ESBL-producing Escherichia coli and Klebsiella pneumoniae: a cross-sectional study of risk factors and comparative genomics of carriage and clinical isolates
Source: mSphere. 2023 Jun 12;8(4):e00025-23. doi: 10.1128/msphere.00025-23 (PMC10470604; doi:10.1128/msphere.00025-23)
Supplement: Table S2 — Sensitivity analysis. [file msphere.00025-23-s0006.pdf]

Supplementary Table 2. Association between ESBL-producing *E. coli* and *K. pneumoniae* species complex (SC) gastrointestinal carriage among 2,973 participants in Tromsø7.

Characteristics	% (ESBL-*E. coli*)	n (ESBL-*E. coli*)	N	AOR	95% CI	p-value
Age (years)						0.123
40-49	2.3	8	344	1.00		
50-59	4.4	19	434	1.86	0.80-4.34	
60-69	3.0	38	1,286	1.02	0.47-2.25	
70-84	2.8	25	909	0.90	0.39-2.10	
Hospitalization past 12 months						0.056
No	2.9	75	2,587	1.00		
Yes	4.3	15	352	1.78	0.99-3.20	
Antibiotic use past 14 days[a]						0.553
No	3.0	85	2,862	1.00		
Yes	4.9	5	103	1.37	0.48-3.90	
Acid suppressive medication past 4weeks						0.335
Not used	2.7	61	2,219	1.00		
≤week	3.2	8	252	1.16	0.54-2.48	
Every week, but not daily	4.1	6	148	1.52	0.64-3.64	
Daily	5.1	9	176	1.89	0.90-3.97	
Travel abroad past 12 months[b]						0.003
No	2.4	31	1,266	1.00		
Other regions (excl. Asia)	2.8	37	1,319	1.24	0.74-2.05	
Asia exclusively + other regions	7.0	19	272	2.96	1.56-5.60	
Traveler`s diarrhea past 12 months[c]						0.599
No	3.1	86	2,805	1.00		
Yes	3.0	3	100	0.72	0.22-2.43	
K. pneumoniae SC gastrointestinal carriage						0.059
No	2.7	67	2,489	1.00		
Yes	4.8	23	484	1.65	0.98-2.77	

ESBL, extended spectrum β-lactamase; N, denominator; AOR, adjusted odds ratio; CI, confidence interval
AOR adjusted for age, hospitalization past 12 months, antibiotic use past 14 days, acid suppressive medication past 4 weeks, travel abroad past 12 months, traveler`s diarrhea past 12 months, and *K. pneumoniae* SC gastrointestinal carriage.

The multivariable model includes 2,651 participants with complete information on all variables.
