# Supplementary material for: Community carriage of ESBL-producing Escherichia coli and Klebsiella pneumoniae: a cross-sectional study of risk factors and comparative genomics of carriage and clinical isolates
Source: mSphere. 2023 Jun 12;8(4):e00025-23. doi: 10.1128/msphere.00025-23 (PMC10470604; doi:10.1128/msphere.00025-23)
Supplement: Table S4 — Comparison of ESBL-E. coli ST131 subclade prevalence. [file msphere.00025-23-s0008.pdf]

Supplementary Table 4. Comparison of ESBL-*E. coli* ST131 subclade prevalence between carriage isolates from Tromsø7 (n=166) and clinical isolates from NORM 2014 (n=118).

ST131 subclade	Tromsø7 % (n)	NORM 2014 % (n)	OR	95%CI	p-value
A	6.0 (10)	13.6 (16)	2.45	1.07-5.60	0.034
B	1.2 (2)	0.8 (1)	0.70	0.06-7.82	0.773
B0	0.6 (1)	0.8 (1)	1.41	0.09-22.78	0.809
C1	11.4 (19)	17.8 (21)	1.67	0.86-3.28	0.132
C2	4.8 (8)	24.6 (29)	6.44	2.82-14.68	<0.001

OR, odds ratio; CI, confidence interval
