# Supplementary material for: Community carriage of ESBL-producing Escherichia coli and Klebsiella pneumoniae: a cross-sectional study of risk factors and comparative genomics of carriage and clinical isolates
Source: mSphere. 2023 Jun 12;8(4):e00025-23. doi: 10.1128/msphere.00025-23 (PMC10470604; doi:10.1128/msphere.00025-23)
Supplement: Table S5 — SNP distances among two putative ESBL-producing E. coli clusters. [file msphere.00025-23-s0009.pdf]

Supplementary Table 5. SNP distances among two putative ESBL-producing *E. coli* clusters identified using a ≤17 SNP cut-off.

Cluster	SNP distance	Sample ID	Collection	Sample material	ST	ST131 subclade
1	4	T7-498	Tromsø7	Feces	ST131	C1
		T7-602	Tromsø7	Feces		
2	6-9	T7-530	Tromsø7	Feces	ST357	-
		T7-549				
		T7-628	Tromsø7	Feces		

SNP, single nucleotide polymorphism
