# Supplementary material for: Community carriage of ESBL-producing Escherichia coli and Klebsiella pneumoniae: a cross-sectional study of risk factors and comparative genomics of carriage and clinical isolates
Source: mSphere. 2023 Jun 12;8(4):e00025-23. doi: 10.1128/msphere.00025-23 (PMC10470604; doi:10.1128/msphere.00025-23)
Supplement: Table S6 — Susceptibility profile of carriage and clinical isolates of ESBL-E. coli ST131 and non-ST131. [file msphere.00025-23-s0010.pdf]

Supplementary Table 6. Susceptibility profile of carriage and clinical isolates of ESBL-*E. coli* ST131 and non-ST131.

	Carriage (Tromsø7)						Clinical (NORM 2014)					
	ST131 (n=40)			non-ST131 (n=126)			ST131 (n=68)			non-ST131 (n=50)		
	%S	%I	%R	%S	%I	%R	%S	%I	%R	%S	%I	%R
Amoxicillin-clavulanic acid i.v.	57.5	-	42.5	57.1	-	42.9	30.9	-	69.1	18.0	-	82.0
Amoxicillin-clavulanic acid*	90.0	-	10.0	90.5	-	9.5	54.4	-	45.6	54.0	-	46.0
Piperacillin-tazobactam	97.5	-	2.5	97.6	-	2.4	70.4†	-	29.6†	64.5†	-	35.5†
Cefuroxime*	0.0	-	100.0	0.8	-	99.2	0.0	-	100.0	4.0	-	96.0
Ceftazidime	17.5	42.5	40.0	21.4	29.4	49.2	13.2	11.8	75.0	6.0	10.0	84.0
Cefotaxime	0.0	2.5	97.5	0.0	2.4	97.6	0.0	0.0	100.0	2.0	2.0	96.0
Cefepime	10.0	60.0	30.0	7.1	53.2	39.7	1.9	7.4	90.7	9.7	3.2	87.1
Ceftazidime-avibactam	100.0	-	0.0	100.0	-	0.0	-	-	-	-	-	-
Ertapenem	100.0	-	0.0	100.0	-	0.0	-	-	-	-	-	-
Meropenem	100.0	0.0	0.0	100.0	0.0	0.0	100.0	0.0	0.0	98.0	2.0	0.0
Aztreonam	5.0	30.0	65.0	5.6	21.4	73.0	-	-	-	-	-	-
Amikacin	100.0	-	0.0	100.0	-	0.0	-	-	-	-	-	-
Gentamicin	70.0	-	30.0	83.3	-	16.7	48.5	-	51.5	48.0	-	52.0
Tobramycin	65.0	-	35.0	80.2	-	19.8	-	-	-	-	-	-
Ciprofloxacin	20.0	10.0	70.0	58.7	15.1	26.2	8.8	11.8	79.4	22.0	4.0	74.0
Trimethoprim-sulfamethoxazole	42.5	0.0	57.5	50.8	0.8	48.4	27.9	0.0	72.1	26.0	0.0	74.0
Nitrofurantoin	97.5	-	2.5	100.0	-	0.0	100.0‡	-	0.0‡	89.5‡	-	10.5‡
Fosfomycin	100.0	-	0.0	99.2	-	0.8	-	-	-	-	-	-
Fosfomycin*	97.5	-	2.5	96.0	-	4.0	-	-	-	-	-	-
Colistin	100.0	-	0.0	98.4	-	1.6	-	-	-	-	-	-
Tigecycline	100.0	-	0.0	100.0	-	0.0	100.0†	-	0.0†	100.0†	-	0.0†

S, susceptible; I, susceptible, increased exposure; R, resistant; i.v. intravenous; *Breakpoints for uncomplicated urinary tract infections; †available for blood culture isolates only (n=85); ‡available for urine culture isolates only (n=33)
